# Prognostic value of gross tumour volume in laryngeal cancer: a systematic review and meta-analysis

**DOI:** 10.1017/S0022215126105313

**Published:** 2026-09

**Authors:** Shahid Iqbal, Saad Illahi, Rumail Zaheer, Ashir Iqbal, Ely Cheikh Boussaty, Charles S. Coffey

**Affiliations:** 1 Department of Otolaryngology - Head & Neck Surgery, University of California San Diego, USA; 2 Calderdale and Huddersfield NHS Foundation Trust, UK; 3 Macquarie University Hospital, Australia; 4 Faculty of Dentistry, Oral & Craniofacial Sciences, King's College London, UK

**Keywords:** Larynx, Laryngeal Neoplasms, Prognosis, Survival, Meta-Analysis

## Abstract

**Objectives:**

Gross tumour volume has been proposed as a complementary prognostic marker in laryngeal squamous cell carcinoma. This systematic review and meta-analysis evaluates the prognostic value of pre-treatment gross tumour volume.

**Methods:**

MEDLINE, Embase and Scopus were systematically searched according to the Preferred Reporting Items for Systematic Reviews and Meta-Analyses guidelines. Studies reporting pre-treatment gross tumour volume and survival outcomes in laryngeal cancer were included. The primary outcome was overall survival, and the secondary outcome was disease-free survival. Hazard ratios were pooled using random-effects meta-analysis. Risk of bias was assessed using the Quality in Prognosis Studies tool, and certainty of evidence using the Grading of Recommendations Assessment, Development and Evaluation approach.

**Results:**

Eight retrospective studies including 1351 patients were included. Increasing gross tumour volume was significantly associated with worse overall survival (pooled hazard ratio per 1 cm^3^ increase: 1.03; 95 per cent confidence interval: 1.02–1.05), with a similar association observed for disease-free survival. Overall risk of bias was moderate.

**Conclusions:**

Higher gross tumour volume is independently associated with poorer survival in laryngeal cancer and may provide prognostic information beyond tumour-node-metastasis staging.

## Introduction

Laryngeal cancer is the second most prevalent head and neck cancer (HNC), with squamous cell carcinoma (SCC) representing the most common histological subtype.^1^ It is a significant cause of morbidity and mortality worldwide, with over 100 000 deaths globally and an estimated incidence of 200 000 cases reported in 2022.^1^ Despite advancements in chemotherapy, radiotherapy and organ-preservation surgical techniques, survival outcomes remain highly variable between patients.^2^ Both the disease itself and its treatment can result in significant impairment of essential functions, including speech, swallowing and breathing.^3^ As a result, laryngeal cancer is associated with considerable physical disability, alongside high levels of psychological morbidity and reduced health-related quality of life. Accurate diagnostic and prognostic stratification is therefore invaluable to inform clinician and patient decision making.

Current prognostication strategies for laryngeal cancer primarily rely on the tumour-node-metastasis (TNM) classification system, which is widely used to estimate survival following treatment.^4^ While TNM staging provides an anatomical assessment of disease extent, it does not account for tumour size in three dimensions. Consequently, tumours with substantially different volumes may be categorised within the same T stage, despite having markedly different prognoses.^5^ Relative to oncological considerations in other head and neck sites, the larynx is anatomically unique in several respects. Tumours can expand into the airway to achieve large volumes without a corresponding increase in TNM stage. Tumour growth may also be confined by rigid anatomical structures such as the thyroid cartilage or defined by invasion through cartilage in ways that impact prognosis out of proportion to tumour volume. Even relatively low-volume tumours may portend poor functional and oncological prognosis if critical locations such as the paraglottic space are involved. This disconnect between T classification and tumour burden has been historically recognised with the salient concept of ‘low-volume T4’^6^ disease, despite a lack of standardised definition of the term. Additionally, this limitation contributes to the observed heterogeneity in overall survival (OS) and local disease control among patients with similar TNM stage, and has prompted increasing interest in quantitative imaging-derived biomarkers (QIBs). This supports an anatomical rationale for evaluating tumour volume as a complementary prognostic marker in laryngeal cancer.

Previous systematic reviews and meta-analyses have suggested a relationship between increasing gross tumour volume (GTV) and adverse outcomes in HNC.^7^ However, this association has not been systematically evaluated in laryngeal cancer as a distinct disease entity. This distinction is clinically important given the substantial global burden of laryngeal cancer and its unique functional consequences.

Gross tumour volume is typically defined as the macroscopic extent of the primary tumour in three dimensions, as delineated on imaging modalities such as computed tomography (CT) or magnetic resonance imaging (MRI).^5^ Larger tumour volumes are associated with increased clonogenic cell number, tumour hypoxia and radioresistance, all of which may adversely affect treatment response.^8^ Several studies have suggested that increased GTV is associated with poorer OS and disease-free survival (DFS) in patients with laryngeal cancer. However, these studies demonstrate variability in volume cut-off values, study design and statistical modelling (dichotomised vs continuous data). In contrast, other authors have reported no significant prognostic value for GTV.^9^ This lack of consensus highlights the need for a comprehensive and systematic evaluation of the available evidence.

The objective of this study was to systematically review the literature and perform a meta-analysis to evaluate the prognostic value of pre-treatment GTV for OS and DFS in patients with laryngeal cancer.

## Materials and methods

The protocol for this systematic review and meta-analysis was prospectively registered with the International Prospective Register of Systematic Reviews (PROSPERO) (CRD420251245243). This study was conducted with reference to the Preferred Reporting Items for Systematic Reviews and Meta-Analyses (PRISMA) statement (Appendix A).^10^ Ethical approval was not required as this study involved publicly available anonymised data.

### Search strategy

A search was undertaken across three electronic databases, namely: Embase, MEDLINE and Scopus. The searches were conducted with no time restriction using a combination of text words and Medical Subject Headings (MeSH) terms. The full search strategy can be found in Appendix B.

### Study selection

Search results from databases were imported into Rayyan (Rayyan Systems, Cambridge, Massachusetts, USA),^11^ and de-duplication was conducted manually by SIq. Thereafter, two authors (SIq and SaI) independently screened the titles and abstracts to determine eligibility, according to the predefined inclusion and exclusion criteria. Following this, full-text screening was conducted independently by the same authors to ensure eligibility for the meta-analysis. Any differences were addressed by consensus.

Eligible studies included retrospective or prospective cohort studies of patients with histopathologically confirmed squamous cell laryngeal carcinoma, in whom GTV was quantified using cross-sectional imaging and linked to OS. Gross tumour volume was defined as the volume of the primary tumour only, excluding involved cervical lymph nodes.

Only studies reporting multivariable survival analyses were included. Studies reporting univariate analyses alone were excluded, as GTV is intrinsically correlated with TNM staging. Unadjusted analyses therefore provide limited incremental prognostic information beyond established staging systems and primarily reiterate the known association between advanced stage and worse outcomes. Studies without a full text in English were excluded.

Studies reporting hazard ratios (HRs) for GTV either as a continuous variable (per cm^3^ increase) or based on a data-driven cut-off value derived using objective methods were eligible for inclusion. Studies that employed arbitrary or non-data-driven GTV cut-off thresholds were excluded.

### Study quality assessment

Two investigators (SIq and SaI) independently assessed the risk of bias of included studies using the Quality In Prognosis Studies (QUIPS) tool.^12^ Each study was graded as having low, moderate, or high risk of bias across the six QUIPS domains, followed by an overall risk-of-bias judgement. Risk-of-bias assessments are summarised in tabular form and visualised using the Risk-of-Bias VISualization (Robvis) tool.^13^

Certainty of evidence was evaluated using the modified Grading of Recommendations Assessment, Development and Evaluation (GRADE) approach for prognostic studies.^14^

### Data synthesis and statistical analysis

Data extracted from each included study comprised first author, year of publication, number of included patients, imaging modality used for GTV measurement, treatment modality, disease stage according to conventional TNM classification and reported HRs for OS and DFS. Data extraction was performed independently by two investigators (SIq and SaI), with discrepancies resolved by consensus.

The primary outcomes of interest were OS and DFS. Overall survival and DFS were treated as time-to-event outcomes. Overall survival was defined as the time from the date of diagnosis or start of treatment to death by any cause. Disease-free survival was defined as the time from treatment until the recurrence of disease or death. Hazard ratios and corresponding 95 per cent confidence intervals (CIs) were extracted directly from the included studies. Where both univariate and multivariable analyses were reported, only HRs derived from multivariable analyses were extracted. An HR greater than 1 was interpreted as indicating worse survival, whereas an HR less than 1 indicated improved survival.

Statistical pooling was performed using Review Manager (RevMan 5.4.1, Cochrane, London, UK).^15^ Hazard ratios were log-transformed prior to meta-analysis, and pooled effect estimates are presented as pooled HRs with 95 per cent CIs, calculated using the inverse-variance method. Given the observational nature of the included studies, a random-effects model was used. Statistical heterogeneity was assessed using the *I*^2^ statistic. Statistical significance was defined at *p* < 0.05.

## Results

### Search results and studies description

**Figure 1.**
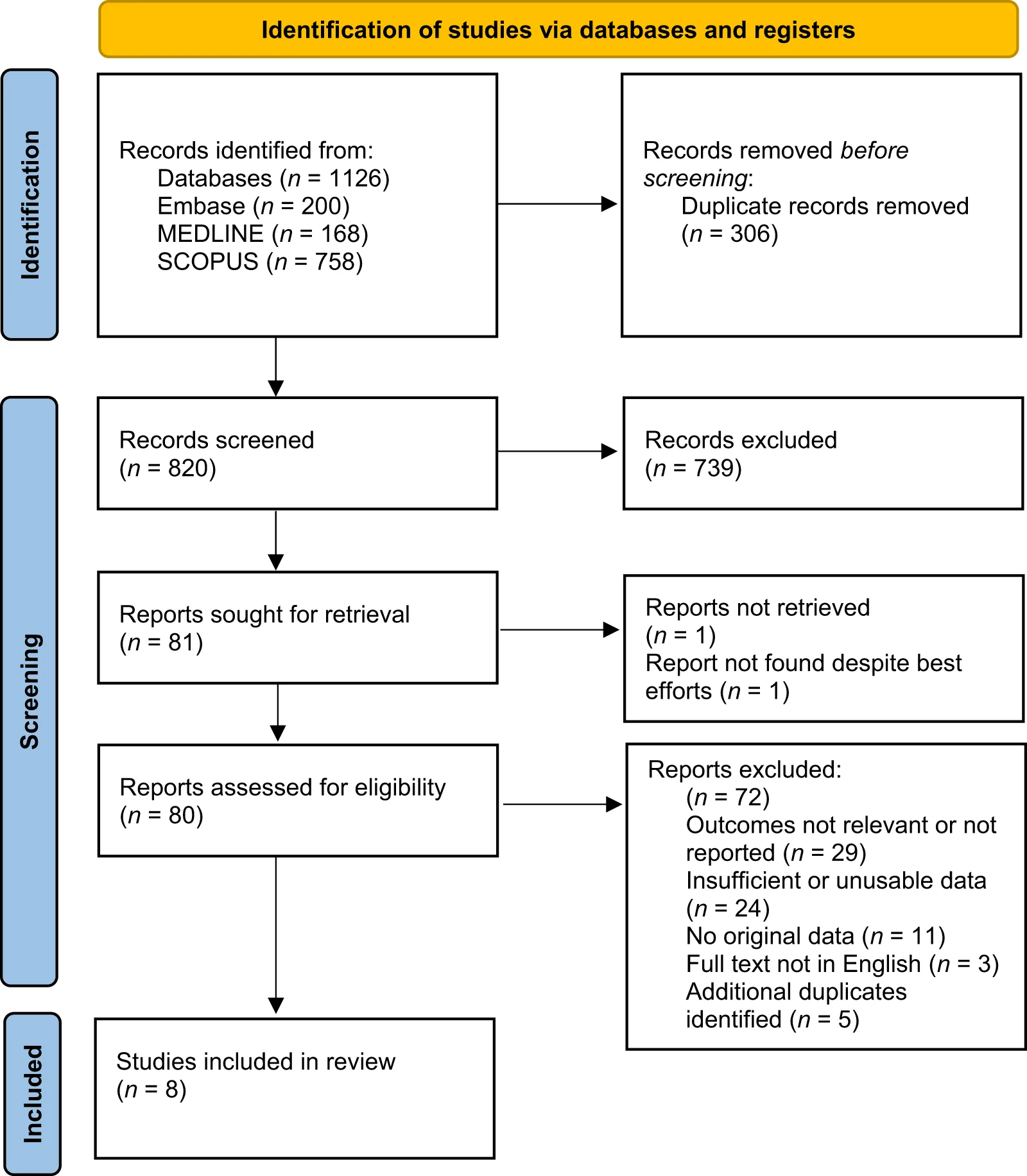
PRISMA flow diagram for systematic search and screening. Figure 1 long description.

Source: Page MJ, et al. BMJ 2021;372:n71. doi: 10.1136/bmj.n71. This work is licensed under CC BY 4.0. To view a copy of this licence, visit https://creativecommons.org/licenses/by/4.0/

The search retrieved 1126 studies. After manual deduplication, 820 studies remained. After title and abstract screening, 81 studies were included for full-text screening. Upon full-text review, 73 studies were excluded, while 8 studies reporting independent patient populations remained in the final inclusion.^16^^–^^23^ Of the studies included, six studies^16^^–^^21^ reported GTV as a continuous variable, and two studies^22^^,^^23^ reported a data-derived dichotomised GTV. Reasons for exclusion and a flow chart of the identification process are shown in Figure 1.

Across the included studies, 1351 patients were included. All patients had their disease confirmed on histopathology. Across included studies which reported GTV, this ranged from 0.20 cm^3^ to 166 cm^3^. Information about sublocation of the tumour was reported by 7 studies, with 49.21 per cent (619) located in the epiglottis, 39.19 per cent (493) located in the glottis, 0.64 per cent (8) located in the subglottis and 10.97 per cent (138) being transglottic. T staging ranged from T1 to T4b, while N staging ranged from N0 to N3 (Table 1).

**Table 1.** Key characteristics of included studies; GTV = gross tumour volume; CT = computed tomography; MRI = magnetic resonance imaging Table 1 long description.

Author, Year	Country	Number	Staging	Treatment	Imaging technique	Range of GTV (cm^3^)	GTV treatment
Malik *et al*., 2023	Canada	319	T3; N0−3; M0	Radiotherapy with or without chemotherapy	CT	0.20−26.96	Continuous
Malik *et al*., 2024	Canada	239	T3; N0–N3; M0	Radiotherapy with or without chemotherapy	CT	0.35−103.11	Continuous
Hanubal *et al*., 2023	USA	93	T1–T4b; N0–N3; M0	Surgery	CT	1.8−166	Continuous
Timmermans *et al*., 2016	Netherlands	166	T3–T4; N0–N1; M0	Surgery and/or chemotherapy and/or radiotherapy	CT/MRI	5.7−21.3	Continuous
Rutkowski *et al*., 2013	Poland	160	T2; N0−2; M0	Radiotherapy	CT	0.15−21.68	Continuous
Rutkowski *et al*., 2014	Poland	105	T2; N0−2; M0	Radiotherapy	CT	–	Continuous
De Andrade *et al*., 2020	Brazil	145	T3–T4a; N0−3	Surgery	CT	–	Dichotomous
Shiao *et al*., 2017	USA	124	T4a; N0−3	Surgery and/or chemotherapy and/or radiotherapy	CT	–	Dichotomous

All included patients were treated with curative intent using definitive radiotherapy alone, or radiotherapy with concurrent chemotherapy, or primary surgical resection with or without adjuvant radiotherapy or chemoradiotherapy. Chemotherapy, when administered, was used exclusively as an adjunct (concurrent or induction) and was not delivered as standalone therapy. In the course of their treatment, 35.38 per cent (478) of patients received surgery, 28.65 per cent (387) of patients received chemotherapy, and 82.38 per cent (1113) of patients received radiotherapy.

Seven studies used CT to delineate GTV, while one study used CT or MRI for GTV delineation. Only one study explicitly reported blinding of the reporting radiologist.

Six studies reported a median follow-up ranging from 19.2 months to 58.8 months, with reported overall range for the studies that did report this from less than 1 month to 159 months.

### Overall survival

**Figure 2.**
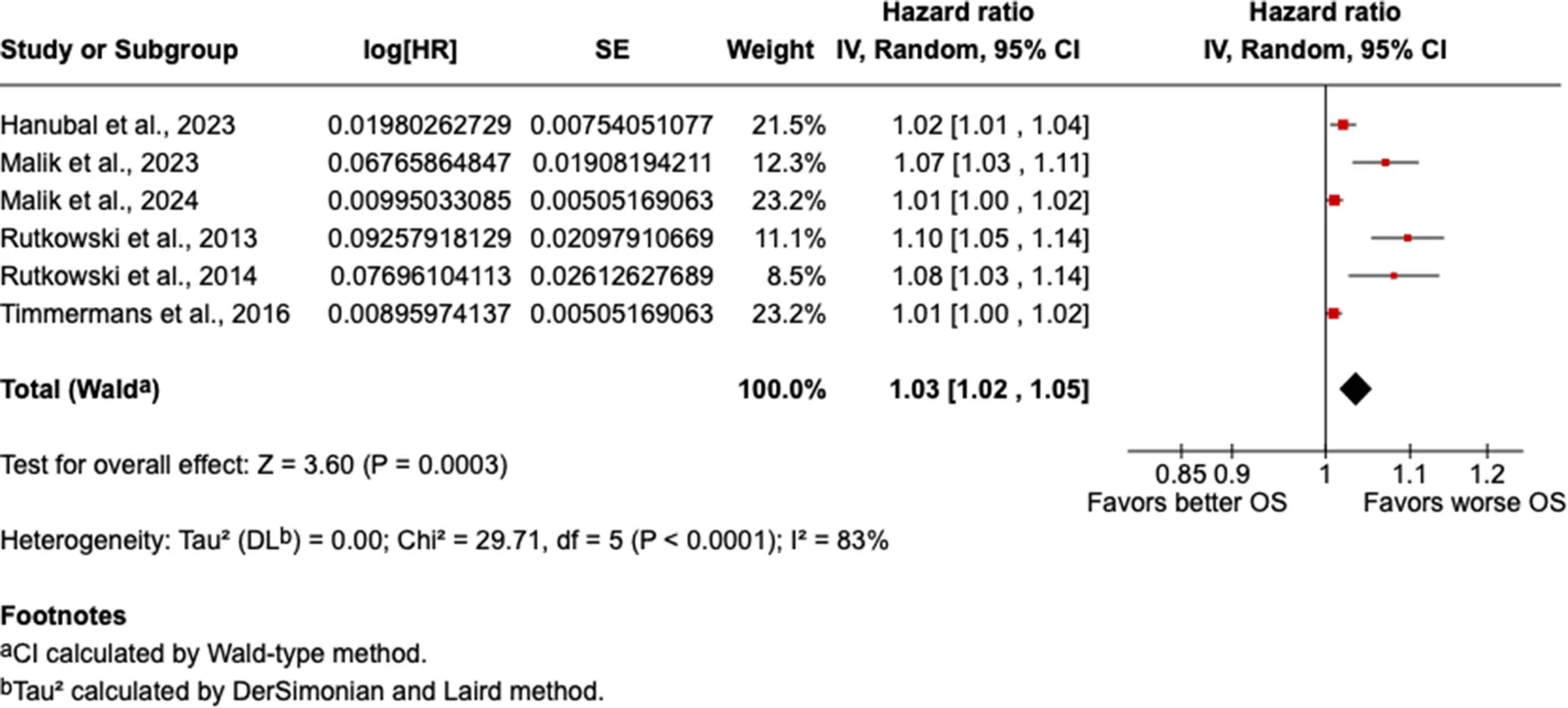
Forest plot of the association between gross tumour volume and overall survival in laryngeal cancer. Figure 2 long description.

Overall survival was analysed by pooling 6 studies,^16^^–^^21^ which reported multivariate analysis of HR based on GTV treated as a continuous variable, comprising 1082 patients. Meta-analysis of these six studies demonstrated a statistically significant association between tumour volume and OS. Using a random-effects model, increasing tumour volume was associated with worse OS (pooled HR = 1.03, 95 per cent CI 1.02–1.05; *p* = 0.0002). However, substantial between-study statistical heterogeneity was observed (*I*^2^ = 83 per cent, *p* < 0.0001) (Figure 2).

### Disease-free survival

**Figure 3.**
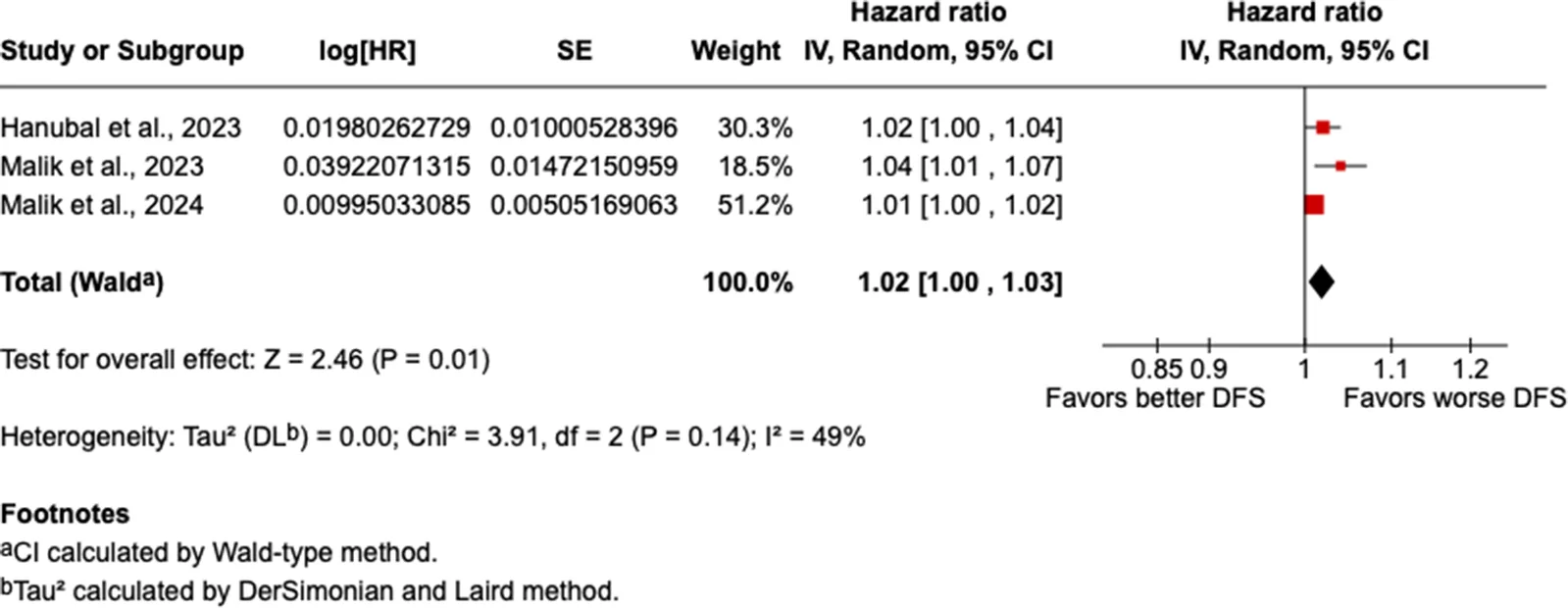
Forest plot of the association between gross tumour volume and disease-free survival in laryngeal cancer. Figure 3 long description.

Disease-free survival was analysed as a secondary outcome by pooling the included studies which reported multivariate analysis of HR based on GTV treated as a continuous variable, comprising 651 patients. Three studies^16^^–^^18^ were included in the meta-analysis for DFS. Increasing tumour volume was associated with worse DFS in a random-effects model (pooled HR = 1.02, 95 per cent CI 1.00–1.03; *p* = 0.01). Moderate statistical heterogeneity was observed across studies (*I*^2^ = 49 per cent, *p* = 0.14) (Figure 3).

**Figure 4.**
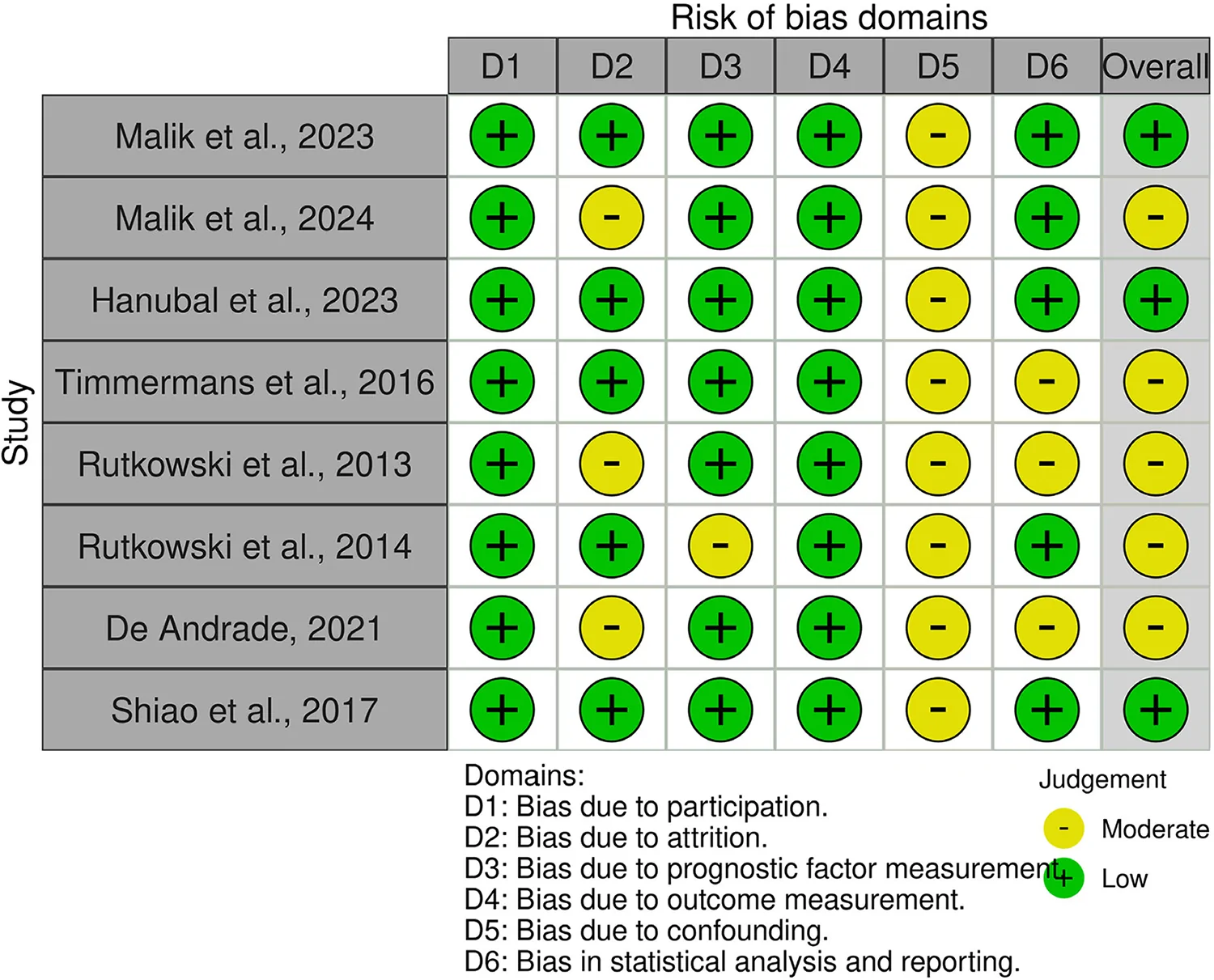
Risk of bias assessment of included studies using the QUIPS tool. Figure 4 long description.

### Qualitative analysis

Two included studies evaluated the prognostic value of GTV using dichotomised thresholds. However, quantitative pooling was not appropriate because the studies derived different cut-off values, resulting in non-comparable HRs. Their findings were therefore synthesised qualitatively.

In a retrospective cohort of surgically treated T3–T4a laryngeal SCC, de Andrade *et al*.^23^ derived an optimal GTV cut-off of 14.2 cm^3^ using receiver operating characteristic curve analysis. Gross tumour volume greater than or equal to 14.2 cm^3^ was associated with worse OS and DFS on univariate analysis; however, GTV did not remain an independent prognostic factor in multivariable Cox regression for either outcome (OS: HR 2.01, 95 per cent CI 0.89–4.57; DFS: HR 2.26, 95 per cent CI 0.52–3.32).

In contrast, Shiao *et al*.^22^ studied patients with T4a laryngeal cancer and used recursive partitioning analysis to identify an optimal GTV threshold of greater than or equal to 21 cm^3^, above which tumour volume was independently associated with significantly worse OS (HR 2.9, 95 per cent CI 1.6–5.2).

Differences in GTV cut-offs and derivation methods limited comparability across the two studies.

### Methodological quality and risk of bias

All included studies were retrospective studies. Risk of bias according to QUIPS was low for three studies and moderate for five studies. There were no studies with a high risk of bias. Moderate risk of bias was mainly due to the inherent risk of uncontrolled confounders in retrospective studies (Figure 4).

### Certainty of evidence

Using a non-contextualised GRADE approach for prognostic factors, the certainty of evidence was rated as moderate for both OS and DFS, downgraded for serious indirectness due to heterogeneity in laryngeal subsites, tumour stages and treatment strategies across included studies (Table 2 and 3).

**Table 2.** GRADE prognosis evaluation of overall survival (OS); CI= confidence interval; GTV = gross tumour volume Table 2 long description.

Domain	Judgement	Explanation
Study design	Observational (cohort studies)	All contributing studies retrospective
Risk of bias	Not serious	QUIPS assessment showed low–moderate risk
Inconsistency	Serious	Substantial statistical heterogeneity (*I*^2^ = 83%)
Indirectness	Serious	Heterogeneity in laryngeal cancer subsites and treatment modalities
Imprecision	Not serious	Narrow CI (1.02−1.05)
Publication bias	Undetected	Unable to assess due to small number of studies
Effect size	Small but consistent	Consistent direction across studies
Dose response gradient	Present	Increasing GTV associated with worse OS
Overall certainty of evidence	MODERATE	Downgraded for indirectness

**Table 3.** GRADE prognosis evaluation of disease-free survival (DFS); CI = confidence interval; GTV = gross tumour value Table 3 long description.

Domain	Judgement	Explanation
Study design	Observational (cohort studies)	All contributing studies retrospective
Risk of bias	Not serious	QUIPS assessment showed low–moderate risk
Inconsistency	Not serious	Moderate statistical heterogeneity (*I*^2^ = 49%)
Indirectness	Serious	Heterogeneity in laryngeal cancer subsites and treatment modalities
Imprecision	Not serious	Narrow CI (1.00−1.03)
Publication bias	Undetected	Unable to assess due to small number of studies
Effect size	Small but consistent	Consistent direction across studies
Dose response gradient	Present	Increasing GTV associated with worse DFS
Overall certainty of evidence	MODERATE	Downgraded for indirectness

## Discussion

To our knowledge, this is the first systematic review and meta-analysis to quantitatively evaluate the prognostic value of GTV in laryngeal cancer. The findings indicate that increasing primary tumour volume is independently associated with worse OS and DFS. Specifically, for each 1 cm^3^ increase in GTV, OS decreases by 3 per cent, while DFS decreases by 2 per cent. These results support GTV as a clinically meaningful prognostic marker in laryngeal cancer. Results for OS and DFS were marked by significant and moderate statistical heterogeneity; however, this is attributable to the different treatment modalities and anatomical subsites implicated across studies.

A major strength of this study lies in its methodology. By restricting inclusion to studies reporting multivariable survival analyses, we specifically assessed the independent prognostic contribution of GTV. This approach avoids repeating the recognised association between advanced TNM stage and adverse outcomes; instead, we managed to isolate the incremental effect of GTV on OS and DFS. Furthermore, the inclusion of patients managed with surgery, radiotherapy, chemotherapy and multimodality treatment reflects clinical practice and enhances the external validity of the findings.

The observed association between tumour volume and outcome is biologically plausible. Larger tumour volumes are likely to reflect increased clonogenic burden^24^ and tumour hypoxia^25^, which have both been linked to poorer outcomes. Furthermore, the infiltrative growth pattern of laryngeal cancers, which commonly involves invasion of the thyroid cartilage, is associated with reduced radiosensitivity compared with exophytic tumours.^26^ These biological considerations provide mechanistic support for the consistent direction of effect observed across included studies.

An additional consideration is the method by which GTV is delineated. The two most commonly used imaging modalities for volumetric assessment are CT and MRI, and there remains debate in the literature regarding the optimal modality for GTV delineation. Smits *et al*.^27^ reported that anatomically based MRI delineation may substantially overestimate tumour volume, with increases of up to 95 per cent compared with CT-based contours. In contrast, Stieb *et al*.^28^ concluded that CT remains the current standard for GTV delineation, citing its rapid acquisition, superior visualisation of high atomic number materials and uniform geometric accuracy across the imaging field.^28^ Other studies have reported no significant differences in GTV delineation between CT and MRI and have advocated for multimodality imaging to improve volumetric accuracy.^29^ Despite this ongoing debate, the majority of studies included in the present review employed CT-based volumetric assessment, providing a relatively consistent methodological approach and thereby strengthening the internal validity of the pooled findings. However, given the difficulty in measuring very small tumours on CT scan, most included patients had advanced disease (T3–T4). Additionally, CT-based measurement of very small laryngeal tumours was limited by poor contrast differentiation, which made distinguishing superficial tumour from adjacent vocalis muscle difficult. Consequently, included studies employed standardised contouring strategies by involving the volume of the adjacent true vocal fold. In two studies^19^^,^^21^, the investigators used endoscopic laryngeal examination to supplement volume delineation.

Inter-observer variability in tumour delineation represents an additional source of uncertainty in volumetric assessment and warrants consideration. In radiation oncology, substantial variability in GTV contouring has been reported, with some studies demonstrating inter-observer standard deviations of up to 60 per cent.^30^ In the present review, GTV delineation was performed by different imaging specialists, and blinding to clinical outcomes was inconsistently reported. These factors may have introduced measurement variability and potential bias. Nevertheless, such limitations reflect clinical practice and highlight the need for standardised contouring protocols and improved reporting of blinding in future studies.

The findings of this systematic review and meta-analysis are consistent with evidence from the broader HNC literature. In a meta-analysis of 15 studies comprising 2447 patients, Xie *et al*.^7^ demonstrated that GTV was a significant prognostic factor for local control (LC), OS and DFS in HNC, with larger GTV associated with worse DFS and OS.

Laryngeal cancer is associated with profound functional morbidity, including loss of speech, impaired swallowing and airway compromise, consequences that are often more severe than those observed in other head and neck subsites. As a result, treatment strategies are predominantly organ-preservation-focused, incorporating radiotherapy, chemotherapy and larynx-preserving surgical approaches, with total laryngectomy reserved for selected cases.^31^ Accurate volumetric risk stratification may therefore be particularly valuable in guiding organ-preserving treatment decisions and balancing oncological control with functional outcomes.

Despite these strengths, several limitations should be acknowledged. All included studies were retrospective, introducing the potential for residual confounding despite multivariable adjustment. Additionally, there was heterogeneity in tumour subsites, disease stages, and treatment strategies across studies, which contributed to indirectness and limited the applicability of pooled estimates to specific clinical scenarios. Variability in imaging modality, contouring methodology and reporting of blinding further highlights the lack of standardisation in assessment of GTV.

Another important limitation relates to the way GTV was modelled. While most included studies treated tumour volume as a continuous variable, two studies employed dichotomised thresholds derived using different data-driven methods. These approaches yielded non-comparable cut-off values and did not allow pooling. This highlights the limitations of dichotomising continuous prognostic variables to evaluate the prognostic value of laryngeal cancer, though this approach could be valuable in determining the implementation of GTV in clinical decision making.

At present, it is unclear whether a universal GTV threshold exists, whether subsite-specific cut-offs are required, or whether tumour volume should be incorporated as a continuous variable into multivariable risk prediction models. Many have even argued for the inclusion of GTV into the TNM staging itself to increase prognostic accuracy.^32^ Addressing these questions will require prospective studies or individual patient data meta-analyses with clear volumetric definitions, grouped by anatomical subsite.

In conclusion, this systematic review and meta-analysis demonstrates that increasing GTV is independently associated with worse survival outcomes in laryngeal cancer, with moderate certainty of evidence. Gross tumour volume represents a valuable prognostic factor; however, further work is needed to standardise its measurement and determine how best to integrate this data into clinically actionable risk stratification frameworks.

## Appendix A

*From:* Page MJ, McKenzie JE, Bossuyt PM, Boutron I, Hoffmann TC, Mulrow CD, et al. The PRISMA 2020 statement: an updated guideline for reporting systematic reviews. BMJ 2021;372:n71. doi: 10.1136/bmj.n71. This work is licensed under CC BY 4.0. To view a copy of this license, visit https://creativecommons.org/licenses/by/4.0/

**Table:**

Section and Topic	Item #	Checklist item	Location where item is reported
**TITLE**			
Title	1	Identify the report as a systematic review.	Title
**ABSTRACT**			
Abstract	2	See the PRISMA 2020 for Abstracts checklist.	Seen
**INTRODUCTION**			
Rationale	3	Describe the rationale for the review in the context of existing knowledge.	Introduction
Objectives	4	Provide an explicit statement of the objective(s) or question(s) the review addresses.	Introduction
**METHODS**			
Eligibility criteria	5	Specify the inclusion and exclusion criteria for the review and how studies were grouped for the syntheses.	Methods
Information sources	6	Specify all databases, registers, websites, organisations, reference lists and other sources searched or consulted to identify studies. Specify the date when each source was last searched or consulted.	Methods
Search strategy	7	Present the full search strategies for all databases, registers and websites, including any filters and limits used.	Methods and appendix
Selection process	8	Specify the methods used to decide whether a study met the inclusion criteria of the review, including how many reviewers screened each record and each report retrieved, whether they worked independently, and if applicable, details of automation tools used in the process.	Methods and PROSPERO registration
Data collection process	9	Specify the methods used to collect data from reports, including how many reviewers collected data from each report, whether they worked independently, any processes for obtaining or confirming data from study investigators, and if applicable, details of automation tools used in the process.	Methods
Data items	10a	List and define all outcomes for which data were sought. Specify whether all results that were compatible with each outcome domain in each study were sought (e.g. for all measures, time points, analyses), and if not, the methods used to decide which results to collect.	Methods
	10b	List and define all other variables for which data were sought (e.g. participant and intervention characteristics, funding sources). Describe any assumptions made about any missing or unclear information.	Methods
Study risk of bias assessment	11	Specify the methods used to assess risk of bias in the included studies, including details of the tool(s) used, how many reviewers assessed each study and whether they worked independently, and if applicable, details of automation tools used in the process.	Methods
Effect measures	12	Specify for each outcome the effect measure(s) (e.g. risk ratio, mean difference) used in the synthesis or presentation of results.	Methods
Synthesis methods	13a	Describe the processes used to decide which studies were eligible for each synthesis (e.g. tabulating the study intervention characteristics and comparing against the planned groups for each synthesis (item #5)).	Methods
	13b	Describe any methods required to prepare the data for presentation or synthesis, such as handling of missing summary statistics, or data conversions.	Methods
	13c	Describe any methods used to tabulate or visually display results of individual studies and syntheses.	Methods
	13d	Describe any methods used to synthesize results and provide a rationale for the choice(s). If meta-analysis was performed, describe the model(s), method(s) to identify the presence and extent of statistical heterogeneity, and software package(s) used.	Methods
	13e	Describe any methods used to explore possible causes of heterogeneity among study results (e.g. subgroup analysis, meta-regression).	NA
	13f	Describe any sensitivity analyses conducted to assess robustness of the synthesized results.	NA
Reporting bias assessment	14	Describe any methods used to assess risk of bias due to missing results in a synthesis (arising from reporting biases).	Methods
Certainty assessment	15	Describe any methods used to assess certainty (or confidence) in the body of evidence for an outcome.	Methods
**RESULTS**			
Study selection	16a	Describe the results of the search and selection process, from the number of records identified in the search to the number of studies included in the review, ideally using a flow diagram.	Results
	16b	Cite studies that might appear to meet the inclusion criteria, but which were excluded, and explain why they were excluded.	Figure 1
Study characteristics	17	Cite each included study and present its characteristics.	Results
Risk of bias in studies	18	Present assessments of risk of bias for each included study.	Results
Results of individual studies	19	For all outcomes, present, for each study: (a) summary statistics for each group (where appropriate) and (b) an effect estimate and its precision (e.g. confidence/credible interval), ideally using structured tables or plots.	Results
Results of syntheses	20a	For each synthesis, briefly summarise the characteristics and risk of bias among contributing studies.	Results
	20b	Present results of all statistical syntheses conducted. If meta-analysis was done, present for each the summary estimate and its precision (e.g. confidence/credible interval) and measures of statistical heterogeneity. If comparing groups, describe the direction of the effect.	Results
	20c	Present results of all investigations of possible causes of heterogeneity among study results.	Discussion
	20d	Present results of all sensitivity analyses conducted to assess the robustness of the synthesized results.	NA
Reporting biases	21	Present assessments of risk of bias due to missing results (arising from reporting biases) for each synthesis assessed.	Results
Certainty of evidence	22	Present assessments of certainty (or confidence) in the body of evidence for each outcome assessed.	Results
**DISCUSSION**			
Discussion	23a	Provide a general interpretation of the results in the context of other evidence.	Discussion
	23b	Discuss any limitations of the evidence included in the review.	Discussion
	23c	Discuss any limitations of the review processes used.	Discussion
	23d	Discuss implications of the results for practice, policy, and future research.	Discussion
**OTHER INFORMATION**			
Registration and protocol	24a	Provide registration information for the review, including register name and registration number, or state that the review was not registered.	Methods
	24b	Indicate where the review protocol can be accessed, or state that a protocol was not prepared.	PROSPERO only
	24c	Describe and explain any amendments to information provided at registration or in the protocol.	NA
Support	25	Describe sources of financial or non-financial support for the review, and the role of the funders or sponsors in the review.	Included
Competing interests	26	Declare any competing interests of review authors.	Included
Availability of data, code and other materials	27	Report which of the following are publicly available and where they can be found: template data collection forms; data extracted from included studies; data used for all analyses; analytic code; any other materials used in the review.	Appendix. Available upon reasonable request

## Appendix B

**Table:**

	Embase search strategy (accessed on December 2^nd^ 2025)
1	‘larynx cancer’/exp OR ‘larynx cancer’
2	(laryn* NEAR/3 (cancer* OR carcinoma* OR tumour* OR tumor*)):ti,ab
3	((glottic OR supraglottic OR subglottic) NEAR/3 (cancer* OR carcinoma*)):ti,ab
4	#1 OR #2 OR #3
5	‘tumor volume’/exp
6	‘tumor burden’/exp
7	‘tumour volume’:ti,ab
8	‘tumor volume’:ti,ab
9	volumetr*:ti,ab
10	((radiologic* OR radiograph*) NEAR/3 volume*):ti,ab
11	((ct OR mri OR pet) NEAR/3 volum*):ti,ab
12	#5 OR #6 OR #7 OR #8 OR #9 OR #10 OR #11
13	‘prognosis’/exp
14	‘survival’/exp
15	prognos*:ti,ab
16	predict*:ti,ab
17	‘overall survival’:ti,ab
18	‘disease free survival’:ti,ab
19	#13 OR #14 OR #15 OR #16 OR #17 OR #18
20	#4 AND #12 AND #19
21	#20 AND english/lim

**Table:**

	MEDLINE search strategy (accessed on December 2^nd^ 2025)
1	Laryngeal Neoplasms/
2	(laryn* adj3 (cancer* or neoplasm* or carcinoma* or tumour* or tumor*)).ti,ab.
3	(glottic adj3 (cancer* or carcinoma*)).ti,ab.
4	(supraglottic adj3 (cancer* or carcinoma*)).ti,ab.
5	(subglottic adj3 (cancer* or carcinoma*)).ti,ab.
6	1 or 2 or 3 or 4 or 5
7	Tumor Burden/
8	“tumour volume”.ti,ab.
9	“tumor volume”.ti,ab.
10	volumetric*.mp. or “volumetry”.ti,ab.
11	radiologic* adj3 volume*.ti,ab.
12	radiograph* adj3 volume*.ti,ab.
13	(“CT volum*” or “MRI volum*” or “PET volum*”).mp.
14	7 or 8 or 9 or 10 or 11 or 12 or 13
15	Prognosis/
16	survival/
17	(prognos* or predict*).mp. or “outcome*”.ti,ab.
18	“disease-free survival”.mp. or “overall survival”.ti,ab.
19	15 or 16 or 17 or 18
20	6 and 14 and 19
21	limit 20 to (english language and humans)

**Table:**

Scopus search strategy (accessed on December 2^nd^ 2025)
(TITLE-ABS-KEY (“laryngeal cancer” OR “laryngeal carcinoma” OR larynx OR glottic OR supraglottic OR “head and neck cancer” W/3 larynx)) AND (TITLE-ABS-KEY (tumor* W/5 volume* OR tumour* W/5 volume* OR volumetric* OR volumetry OR “3D measurement” OR “three dimensional” OR segmentation OR radiomic* OR “gross tumor volume” OR GTV)) AND (TITLE-ABS-KEY (prognosis OR survival OR “overall survival” OR “disease-free survival” OR recurrence OR outcome* OR “local control” OR “treatment failure”))

## Figure 1 Long description

The flowchart details the process of identifying studies via databases and registers. In the identification phase, records were identified from databases totaling 1126, with 200 from Embase, 168 from MEDLINE and 758 from SCOPUS. Before screening, 306 duplicate records were removed. In the screening phase, 820 records were screened and 739 were excluded. Reports sought for retrieval numbered 81, with 1 report not retrieved due to being not found despite best efforts. Reports assessed for eligibility were 80, with 72 excluded for reasons such as outcomes not relevant or not reported (29), insufficient or unusable data (24), no original data (11), full text not in English (3) and additional duplicates identified (5). Finally, 8 studies were included in the review.

Navigate back to Figure 1.

## Figure 2 Long description

Hazard ratio, Random, 95 percent CI. A forest plot lists six studies in rows with columns labeled, Study or Subgroup, log open bracket HR close bracket, SE, Weight, IV, Random, 95 percent CI and a rightmost plot of hazard ratio points with horizontal confidence intervals. The rightmost plot has a horizontal scale labeled 0.85, 0.9, 0.95, 1, 1.05, 1.1, 1.2, with text below reading, Favors better OS on the left and Favors worse OS on the right. Row values shown: Hanubal et al., 2023: log open bracket HR close bracket 0.019806262729, SE 0.00754051077, Weight 21.5 percent, hazard ratio 1.02 open bracket 1.01, 1.04 close bracket. Malik et al., 2023: log open bracket HR close bracket 0.06765864847, SE 0.01908491211, Weight 12.3 percent, hazard ratio 1.07 open bracket 1.03, 1.11 close bracket. Malik et al., 2024: log open bracket HR close bracket 0.00995033085, SE 0.00505169063, Weight 23.2 percent, hazard ratio 1.01 open bracket 1.00, 1.02 close bracket. Rutkowski et al., 2013: log open bracket HR close bracket 0.09257918129, SE 0.02097910669, Weight 11.1 percent, hazard ratio 1.10 open bracket 1.05, 1.14 close bracket. Rutkowski et al., 2014: log open bracket HR close bracket 0.07696104113, SE 0.02612627689, Weight 8.5 percent, hazard ratio 1.08 open bracket 1.03, 1.14 close bracket. Timmermans et al., 2016: log open bracket HR close bracket 0.00895974137, SE 0.00505169063, Weight 23.2 percent, hazard ratio 1.01 open bracket 1.00, 1.02 close bracket. A Total row shows 100.0 percent and a pooled hazard ratio of 1.03 open bracket 1.02, 1.05 close bracket, marked by a diamond on the plot. Text below the table reads: Total open bracket Wald close bracket a. Test for overall effect: Z equals 3.60 open bracket P equals 0.0003 close bracket. Heterogeneity: Tau superscript 2 equals 0.00; Chi superscript 2 equals 29.71, df equals 5 open bracket P less than 0.0001 close bracket; I superscript 2 equals 83 percent. Footnotes: a CI calculated by Wald type method. b Tau superscript 2 calculated by DerSimonian and Laird method.

Navigate back to Figure 2.

## Figure 3 Long description

Study or Subgroup. A table lists four columns: log HR, SE, Weight and Hazard ratio IV, Random, 95 percent CI. Row 1: Hanubal et al., 2023. log HR 0.01980262729. SE 0.01000528396. Weight 30.3 percent. Hazard ratio 1.02, 1.00, 1.04. Row 2: Malik et al., 2023. log HR 0.03922071315. SE 0.01472150959. Weight 18.5 percent. Hazard ratio 1.04, 1.01, 1.07. Row 3: Malik et al., 2024. log HR 0.00995033085. SE 0.00505169063. Weight 51.2 percent. Hazard ratio 1.01, 1.00, 1.02. Total row: Total, Wald a. Weight 100.0 percent. Hazard ratio 1.02, 1.00, 1.03. Text below the table states: Test for overall effect: Z equals 2.46, P equals 0.01. Text below that states: Heterogeneity: Tau superscript 2, DL equals 0.00; Chi superscript 2 equals 3.91, df equals 2, P equals 0.14; I superscript 2 equals 49 percent. A forest plot to the right shows three square markers with horizontal confidence interval lines aligned to the three study rows and a diamond marker aligned to the total row. The horizontal axis is labeled with tick values 0.85, 0.9, 0.95, 1, 1.1 and 1.2. Text under the axis reads, Favors better DFS on the left and Favors worse DFS on the right. Footnotes section shows: a Tau superscript 2 calculated by Wald type method. b Tau superscript 2 calculated by DerSimonian and Laird method.

Navigate back to Figure 3.

## Figure 4 Long description

The table has columns: Study, D1, D2, D3, D4, D5, D6, Overall. Row 1: Malik et al., 2023; low, low, low, low, low, low, low. Row 2: Malik et al., 2024; low, low, low, low, low, low, low. Row 3: Hanubal et al., 2023; low, low, low, low, low, low, low. Row 4: Timmermans et al., 2016; low, low, low, low, low, low, low. Row 5: Rutkowski et al., 2013; low, low, low, low, low, low, low. Row 6: Rutkowski et al., 2014; low, low, low, low, low, low, low. Row 7: De Andrade, 2021; low, low, low, low, low, low, low. Row 8: Shiao et al., 2017; low, low, low, low, low, low, low. Domains are: D1 bias due to participation, D2 bias due to attrition, D3 bias due to prognostic factor measurement, D4 bias due to outcome measurement, D5 bias due to confounding, D6 bias in statistical analysis and reporting. Judgments are indicated by green for low and yellow for moderate.

Navigate back to Figure 4.

## Table 1 Long description

Study characteristics are listed by author and year, country, number of participants, staging, treatment, imaging technique, range of GTV in cubic centimetres, and how GTV was treated. Malik et al., 2023 in Canada included 319 participants with staging T3, N0 to 3, M0; treatment was radiotherapy with or without chemotherapy; imaging was CT; GTV range was 0.20 to 26.96; GTV treatment was continuous. Malik et al., 2024 in Canada included 239 participants with staging T3, N0 to N3, M0; treatment was radiotherapy with or without chemotherapy; imaging was CT; GTV range was 0.35 to 103.11; GTV treatment was continuous. Hanubal et al., 2023 in the USA included 93 participants with staging T1 to T4b, N0 to N3, M0; treatment was surgery; imaging was CT; GTV range was 1.8 to 166; GTV treatment was continuous. Timmermans et al., 2016 in the Netherlands included 166 participants with staging T3 to T4, N0 to N1, M0; treatment was surgery and or chemotherapy and or radiotherapy; imaging was CT and MRI; GTV range was 5.7 to 21.3; GTV treatment was continuous. Rutkowski et al., 2013 in Poland included 160 participants with staging T2, N0 to 2, M0; treatment was radiotherapy; imaging was CT; GTV range was 0.15 to 21.68; GTV treatment was continuous. Rutkowski et al., 2014 in Poland included 105 participants with staging T2, N0 to 2, M0; treatment was radiotherapy; imaging was CT; the GTV range cell is blank; GTV treatment was continuous. De Andrade et al., 2020 in Brazil included 145 participants with staging T3 to T4a, N0 to 3; treatment was surgery; imaging was CT; the GTV range cell is blank; GTV treatment was dichotomous. Shiao et al., 2017 in the USA included 124 participants with staging T4a, N0 to 3; treatment was surgery and or chemotherapy and or radiotherapy; imaging was CT; the GTV range cell is blank; GTV treatment was dichotomous.

Navigate back to Table 1.

## Table 2 Long description

Judgements are listed by domain with an explanation for prognosis evaluation of overall survival. Study design was observational, cohort studies, with all contributing studies retrospective. Risk of bias was judged not serious, with a QUIPS assessment showing low to moderate risk. Inconsistency was judged serious due to substantial statistical heterogeneity, with I2 reported as 83 percent. Indirectness was judged serious due to heterogeneity in laryngeal cancer subsites and treatment modalities. Imprecision was judged not serious, with a narrow confidence interval from 1.02 to 1.05. Publication bias was undetected, with inability to assess due to a small number of studies. Effect size was described as small but consistent, with consistent direction across studies. Dose response gradient was present, with increasing gross tumour volume associated with worse overall survival.

Navigate back to Table 2.

## Table 3 Long description

Rows list GRADE domains with columns for judgement and explanation for disease free survival. Study design is observational, cohort studies, with all contributing studies retrospective. Risk of bias is not serious, with QUIPS assessment showing low to moderate risk. Inconsistency is not serious, with moderate statistical heterogeneity and an I2 value of 49 percent. Indirectness is serious, explained by heterogeneity in laryngeal cancer subsites and treatment modalities. Imprecision is not serious, with a narrow confidence interval from 1.00 to 1.03. Publication bias is undetected, with inability to assess due to a small number of studies. Effect size is small but consistent, with consistent direction across studies. Dose response gradient is present, with increasing gross tumour value associated with worse disease free survival.

Navigate back to Table 3.
